# Recent advances in immunopathogenesis and clinical practice: mastering the challenge-managing of non-tuberculous mycobacteria

**DOI:** 10.3389/fimmu.2025.1554544

**Published:** 2025-03-19

**Authors:** Wiwat Chancharoenthana, Supitcha Kamolratanakul, Suwatchareeporn Rotcheewaphan, Asada Leelahavanichkul, Marcus J. Schultz

**Affiliations:** ^1^ Department of Clinical Tropical Medicine, Faculty of Tropical Medicine, Mahidol University, Bangkok, Thailand; ^2^ Tropical Immunology and Translational Research Unit (TITRU), Department of Clinical Tropical Medicine, Faculty of Tropical Medicine, Mahidol University, Bangkok, Thailand; ^3^ Department of Microbiology, Faculty of Medicine, Chulalongkorn University, Bangkok, Thailand; ^4^ Center of Excellence on Translational Research in Inflammatory and Immunology (CETRII), Department of Microbiology, Chulalongkorn University, Bangkok, Thailand; ^5^ Department of Intensive Care & Laboratory of Experimental Intensive Care and Anesthesiology (L.E.I.C.A), Academic Medical Center, University of Amsterdam, Amsterdam, Netherlands; ^6^ Centre for Tropical Medicine and Global Health, Nuffield Department of Medicine, Oxford University, Oxford, United Kingdom; ^7^ Mahidol–Oxford Tropical Medicine Research Unit, Faculty of Tropical Medicine, Mahidol University, Bangkok, Thailand

**Keywords:** non-tuberculous mycobacteria, m. avium complex, treat, environmental, vulnerable

## Abstract

Non-tuberculous mycobacteria (NTM) are widespread environmental pathogens that can lead to significant disease burden, particularly in immunocompromised individuals, but also in those with a normal immune system. The global incidence of NTM is increasing rapidly, with *Mycobacterium avium* complex (MAC) being one of the most common types. The immunopathogenesis of the MAC involves a complex interaction between the bacteria and the host immune system. MAC survives and replicates within macrophages by preventing the fusion of phagosomes and lysosomes. The mycobacteria can neutralize reactive oxygen and nitrogen species produced by the macrophages through their own enzymes. Additionally, MAC modulates cytokine production, allowing it to suppress or regulate the immune response. Diagnosing MAC infections can be challenging, and the effectiveness of available treatments may be limited due to MAC’s unpredictable resistance to various antimycobacterial drugs in different regions. Treating MAC infection requires a collaborative approach involving different healthcare professionals and ensuring patient compliance. This review aims to shed light on the complexities of MAC infection treatment, discussing the challenges of MAC infection diagnosis, pharmacological considerations, such as drug regimens, drug monitoring, drug interactions, and the crucial role of a multidisciplinary healthcare team in achieving the best possible treatment outcomes for patients.

## 1 Introduction

**Figure 1:**
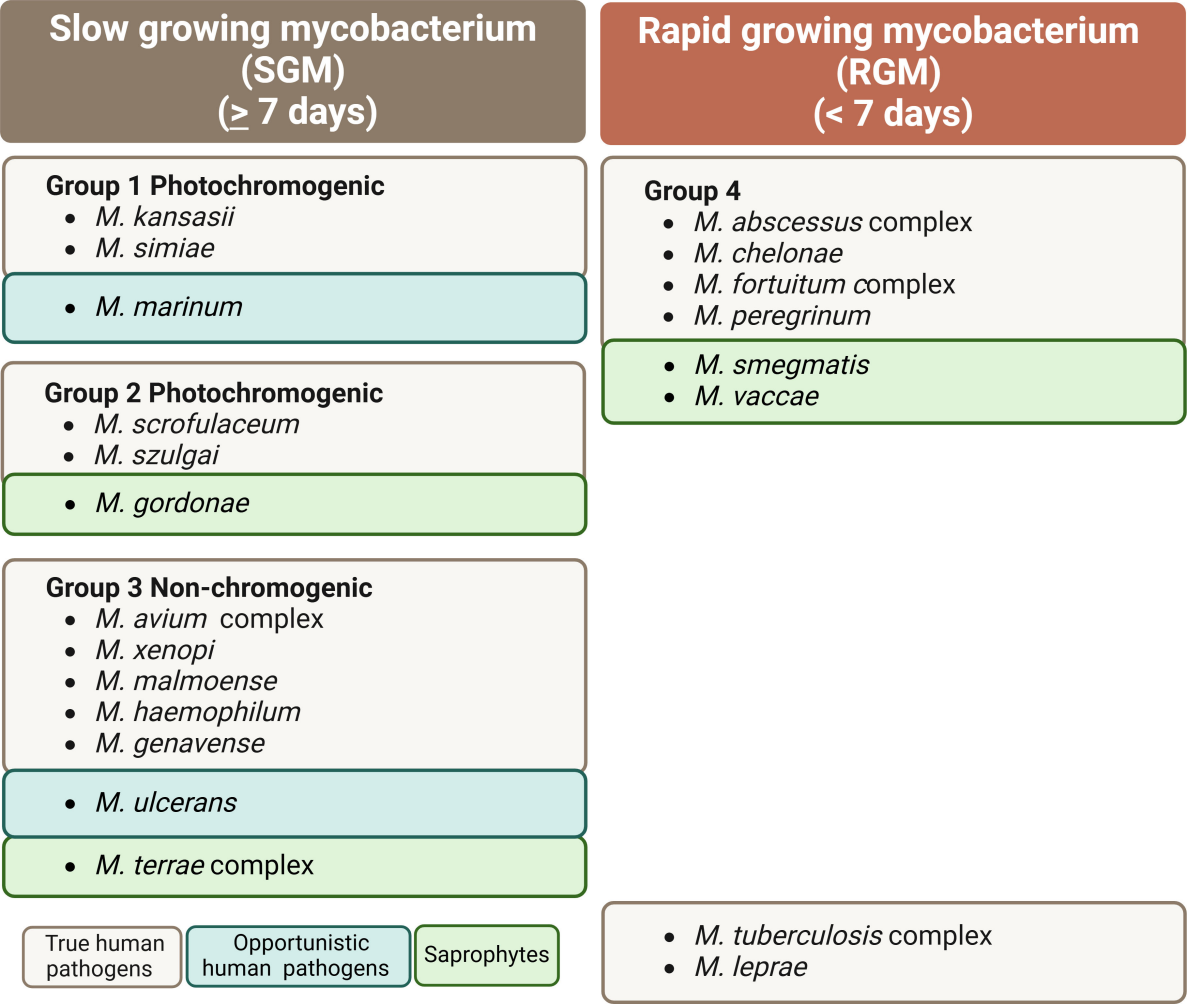
The non-tuberculosis mycobacteria Runyon classification system.

The number of non-tuberculous mycobacteria (NTM) infections is increasing worldwide. Unlike tuberculosis, which is caused by *Mycobacterium tuberculosis* and requires mandatory reporting, NTM infection reporting is not mandatory. This makes it difficult to determine the incidence and prevalence of the different species. NTM are typically found in soil and water and do not include the *M. tuberculosis*, *Mycobacterium leprae*, and *Mycobacterium lepromatosis*. The Runyon classification system (**Figure 1**), which categorizes NTM based on colony appearance, growth rate in media, and pigment production (1–3), is the most widely used classification method, and currently, >200 NTM species have been identified based on genomic criteria (1, 2). *M. avium*, a slow-growing mycobacterium, is associated with *Mycobacterium avium* complex (MAC) infections that can cause chronic pulmonary disease, disseminated disease, as well as lymphadenitis (1).

Recently, NTM infection is becoming more prevalent in the United States, Europe, and other developed countries in the Western world (1). In 22 studies conducted between 1946 and 2014, it was discovered that there was an 81% decrease in tuberculosis cases, while NTM increased by 94% across different regions (4). NTM diseases are increasingly being recognized as causes of opportunistic infections in individuals with severe immune deficiencies, as well as those with congenital or acquired lung conditions, and in healthcare settings (4). The challenges in determining the true incidence and shifting epidemiology continue as we move away from the era of uncontrolled human immunodeficiency virus (HIV). However, we are still seeing an increased in NTM diagnosed in an aging and increasingly immunocompromised population. The incidence and prevalence of NTM infections in individuals with HIV/acquired immunodeficiency syndrome (AIDS) vary based on geographic location, the level of immunosuppression, and access to antiretroviral therapy (ART) (5). Before the widespread use of ART, NTM infections, especially MAC infection, were significant causes of morbidity among HIV/AIDS patients. In the pre-ART era, the incidence of disseminated MAC infection in HIV patients with CD4 counts below 50 cells/μl was estimated to be between 20% and 40% annually (6). Since the advent of ART, the incidence of NTM infections has significantly decreased. Current studies indicate that the incidence of disseminated MAC infection in HIV patients receiving ART is now less than 1% annually (7). In terms of prevalence, during the pre-ART era, the prevalence of disseminated NTM (primarily MAC infection) among advanced HIV/AIDS patients with CD4 counts below 50 cells/μl was reported to be between 10% and 30%. Although the prevalence of NTM infections has dramatically declined in the ART era, localized NTM infections (such as pulmonary NTM) continue to be a concern, particularly in regions with high environmental exposure to NTM (8, 9).

Additionally, there has been a noted rise in NTM infections among individuals who are not immunocompromised and do not have preexisting lung diseases. One study found that 18% of MAC pulmonary infections were in people without predisposing conditions, which is consistent with other literature on this topic (10, 11). Another study revealed that this group accounted for 20% of all clinical NTM infections, and they tended to experience delayed diagnosis and high recurrence rates (12).

While many individuals exposed to NTM show no symptoms, certain NTM species, such as *M. avium*, can lead to severe infections in both immunocompromised and immunocompetent individuals. While high-risk populations, including those with chronic lung disease, HIV/AIDS, anti-interferon-gamma antibody-associated adult-onset immunodeficiency syndrome (AIGAs-AOID), and immunosuppression, are more susceptible to localized or disseminated NTM infections, NTM infections are not limited to these populations (1, 13–17). NTM infections pose significant treatment challenges due to antibiotic resistance.

This review discusses the processes of NTM infection, mainly focusing on MAC and its immunopathogenesis, virulence factors, and host interactions while highlighting the commonalities regarding clinical presentation spectrums in particular populations. Drug resistance mechanisms and potential future therapeutic options are also discussed. Understanding this underrecognized global threat is crucial as it may lead to critical illness.

## 2 Immunopathogenesis and immune response to *Mycobacterium avium* complex

The prevalence of MAC infection in the immunocompromised hosts is high, particularly in skin and soft tissue infections when compared to mycobacteremia, tenosynovitis, and lymphadenopathy (18). This suggests that the host’s immunity is generally protective against MAC in the general population, unless there is a breakdown in barriers or a suitable environment for MAC accumulation and growth. However, the role of MAC and its impact on patients varies significantly depending on the immune status of the host. Notably, in immunocompetent individuals, MAC infections are rare and typically present as localized pulmonary disease. This is especially true for those with pre-existing lung conditions, such as chronic obstructive pulmonary disease (COPD) or bronchiectasis. The infection is usually slow-growing and chronic, with symptoms including cough, fatigue, weight loss, and fever. Disseminated MAC infections are uncommon in immunocompetent hosts (5). In contrast, MAC can cause severe and disseminated disease in immunosuppressed patients. This is particularly the case for individuals with advanced HIV/AIDS (with a CD4 count of less than 50 cells/μl), those undergoing chemotherapy, or organ transplant recipients. In these patients, MAC often spread beyond the lungs and may involve the bloodstream, lymph nodes, bone marrow, liver, and other organs. This condition is referred to as disseminated MAC infection. Before the introduction of ART, disseminated MAC infection was a leading cause of morbidity and mortality in this population (6, 19).

### 2.1 Cellular and innate immune response

Due to NTM infection being less virulent than tuberculosis, several host defense defects are usually presented in the host, including ciliary dysfunction, lung structural changes, pulmonary clearance defects, poor secretion clearance, and immune suppression (20). Unlike tuberculosis, NTM is an environmental microbe found in various reservoirs such as garden soils, water sources, and shower heads (21), and it also colonizes the human respiratory tract (22). However, innate and adaptive immune responses against NTM are different to those against *M. tuberculosis* because NTM presents different pathogen-associated molecular patterns (PAMPs), which are recognized by pathogen-recognition receptors at the plasma membrane of macrophages (23). The CD4 Th1 and myeloid cells, including macrophages, dendritic cells, and neutrophils, play a crucial role in the initial recognition, phagocytosis, and controls of mycobacterial infection and chronic disease (23). In the alveoli and airways, alveolar macrophages are the first-line innate immune cells that detect airborne (bioaerosol) microbes. In contrast, lung interstitial macrophages act at the vasculature and lung interstitial levels, mainly on hematogenous spreading organisms (24).

Once *M. avium* enters the lungs, the alveolar macrophages are the first immune cells to respond. They interact with MAC through fibronectin receptors and complement pathways (25). The membrane of *M. avium* has a high affinity for the fibronectin receptor on macrophages, specifically binding to its mannosyl and fucosyl moieties (26). Alveolar macrophages utilize various pathways to enhance the apparent phagocytic activities, such as phagocytosis by primary phagosomes, which ultimately damage the bacilli through toxic oxygen metabolites, acidification, and neutrophil defensins (27, 28), as well as binding to complement and mannose receptors, surfactant molecules, dendritic cell-specific intracellular adhesion molecule-3-grabbing nonintegrin, and Toll-like receptors (TLR) (29). TLR2 recognizes 19-kDa mycobacterial lipoproteins and glycolipids, whereas TLR2/1 and TLR2/6 heterodimers respond to triacylated and diacylated lipoproteins, respectively. Moreover, heat shock protein 60/65 and mycobacterial unmethylated CpG DNA are sensed by TLR4 and TLR9, respectively (22). Activated macrophages then secrete interleukin (IL)-12, which activates the secretion of interferon (IFN)-γ (the IL-12/IFN-γ axis [an innate and adaptive immunity cross talk]) and IL-17 (from T helper [Th] 17 cells, which also functions as an antimycobacterial cytokine by enhancing IFN-γ-mediated Th1 responses) by natural killer and Th1 cells (30). Innate immunity and Th1 seem to be the primary anti-NTM responses; further studies are needed because host vulnerability and evasion mechanism studies are very few compared to those on anti-*M. tuberculosis* responses. As an antigen presenting cells, macrophages also defend against MAC by presenting it to T lymphocytes, recruiting and cloning specific T lymphocytes, and training immunologic memory T cell expansion.

However, MAC can survive intracellularly in macrophages and proliferate in their vacuoles. This is achieved by inhibiting the fusion of phagosomes with lysosomes, creating a more anaerobic environment for their growth, and inducing MAC genes for duplication. Additionally, MAC inhibits host macrophage function and lymphocyte proliferation, and induces macrophage apoptosis by down-regulating the *Bcl-2* gene product, which functions as an apoptosis inhibitor (31, 32). The dynamic cellular immune responses lead to the formation of granulomas, where clusters are surrounded by mononuclear inflammatory cells and epithelioid histiocytes (33). Notably, the release of cytolytic enzymes and other cytotoxic proteins causes necrosis and encapsulating fibrosis in either affected area or adjacent tissue (33, 34).

**Figure 2:**
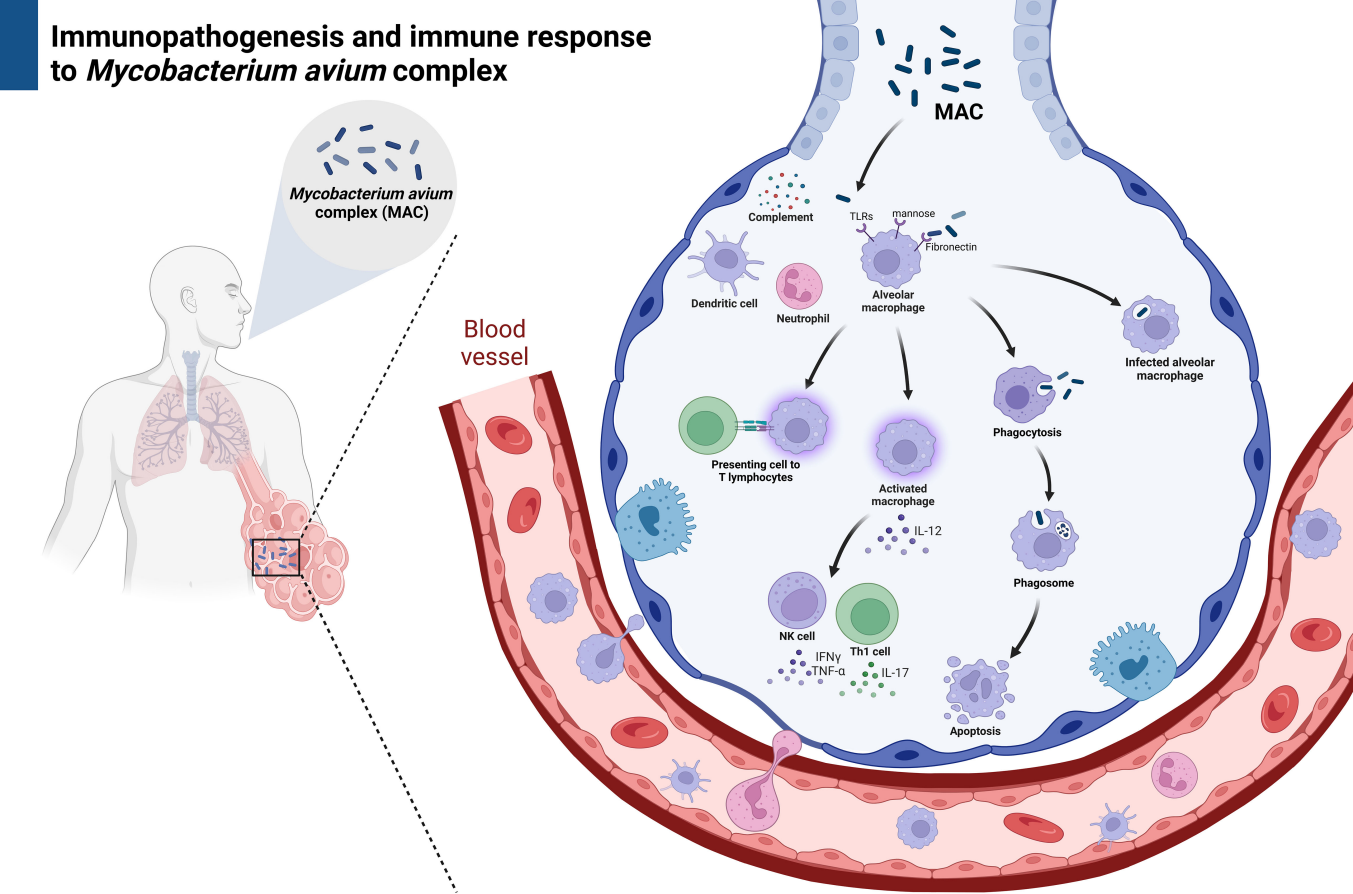
The immune responses against non-tuberculous mycobacteria. Created with BioRender.com.

The cytokine network plays a crucial role in the immune response against MAC, working closely with macrophages and involving T lymphocytes, natural killer (NK) cells, interleukin-12, tumor necrosis factor (TNF)-α, and IFN-γ. Activated macrophages respond by producing IL-12, which in turn activates T lymphocytes and NK cells (35). NK cells respond to MAC-infected cells by secreting TNF-α and IFN-γ. This leads to the activation of neutrophils and other macrophages, producing superoxide and nitric oxide, and increasing the expression of certain molecules (36). Activated macrophages also decrease lysosomal pH, increase intracellular concentration of antimycobacterial agents, and provide positive feedback signals to enhance the mycobactericidal effect (37). Patients with defects in these molecular receptors or related cytokine genes are more susceptible to MAC (38). For example, those with Mendelian susceptibility to mycobacterial diseases (MSMD) have a narrow vulnerability to certain mycobacteria, with recurrent infections characterized by the presence of neutralizing auto-antibodies (39). Furthermore, the use of IFN-α inhibitors in the treatment of rheumatoid arthritis can disrupt the immune response to MAC, likely by inhibiting the macrophage killing ability to MAC and neutralizing circulating IFN-α levels (40, 41). This interruption significantly increases the risk of NTM infection in patients receiving TNF-α blocker treatments (42). IL-12 stimulates T lymphocytes and NK cells to produce IFN-γ and IFN-α, and it is also stimulated by IFN-γ and IFN-α. On the other hand, IL-10 down-regulates the inflammatory response, which makes the host more susceptible to MAC (43, 44) (**Figure 2**). Identifying this novel target would be a future perspective for elucidating a critical pathway for controlling MAC. Indeed, targeting to IL-12 and IFN-γ/IFN-α feedback loop could involve enhancing IL-12 signaling to boost IFN-γ production, developing agonists or mimetics of IL-12 or IFN-γ to strengthen the host’s antimicrobial response, and lastly, identifying and blocking negative regulators of this pathway that may be exploited by MAC to evade immunity (43, 44). Meanwhile, targeting IL-10 could involve developing IL-10 antagonists or blocking its receptor (IL-10R) to reduce its immunosuppressive effects, identifying downstream signaling molecules of IL-10 that could be modulated to restore a balanced immune response, and exploring the role of IL-10-producing regulatory T cells (Tregs) and their contribution to MAC susceptibility (43, 44). However, currently, there is no solid evidence of a suspected target molecule or specific immune deficiency that has been established for advanced treatment.

Interestingly, a recent study from Seto and colleagues (45) demonstrates that while tuberculosis and MAC granulomas share some common features, such as the formation of granulomatous structures, they differ significantly in their molecular and cellular profiles. A strong Th1-driven immune response, active bacterial replication, and caseous necrosis characterize tuberculosis granulomas. In contrast, MAC granulomas exhibit a more subdued immune response, chronic inflammation, and a tendency towards fibrosis. These differences are reflected in their proteomic profiles, metabolic pathways, and histopathological features. The tuberculosis granulomas often exhibit caseous necrosis, surrounded by a rim of epithelioid macrophages, giant cells, and lymphocytes. The granulomas are well-organized and tightly packed, reflecting a strong immune response, whereas the MAC granulomas tend to be less organized, with fewer giant cells and more diffuse inflammation. In addition, the lesions are more likely to be fibrotic and less necrotic, with a higher presence of foamy macrophages (45).

### 2.2 Immune escape of *Mycobacterium avium* complex

The cell wall properties of mycobacteria prevent the acidification of phagocytosis and enhance biofilm formation, enabling these pathogens to escape detection by the human immune system (46, 47). However, the mechanisms responsible for biofilm formation by mycobacteria, which helps them survive antimycobacterial agents, are not yet fully understood. Additionally, MAC inhibits the production of inflammatory cytokines, which helps them evade the host’s immune response and facilitates colonization. Glycopeptidolipids (GPLs) are a class of glycolipids produced and expressed in different forms with production of highly antigenic, typeable serovar-specific GPLs in members of MAC. Smooth-domed colony types induce greater production of inflammatory cytokines, while the smooth-transparent colonial types trigger a weaker host response, producing fewer cytokines (48).

### 2.3 Host susceptibility

#### 2.3.1 Exploring the relationship between body morphotype and preexisting nodular bronchiectasis and fibrocavitary chronic lung diseases

The body morphotype is a predisposing factor for developing chronic lung disease, which in turn increases the risk of developing pulmonary MAC infection, including nodular bronchiectasis disease (NB type) and fibrocavitary disease (FC type). Some patients exhibit common body composition features such as scoliosis, pectus excavatum, mitral valve prolapse, and joint hypermobility. These conditions may be linked to genetic factors associated with MAC infection, although the exact mechanism by which this body morphotype predisposes to MAC infection is unclear. One proposed mechanism involves multigene mutations, including heterozygosity for the cystic fibrosis transmembrane conductance regulator (CFTR) gene mutation, as well as certain connective tissue disorders that contribute to MAC infection, such as Marfan syndrome (49–51), congenital contractual arachnodactyly (52), and complete TYK2 deficiency in hyper-IgE syndrome (53).

In fibrocavitary chronic lung disease, MAC infection is often challenging to distinguish from tuberculosis. This is mainly because it tends to affect the upper lobes of the lungs in elderly, male, and heavy-smoking individuals. It can also present with symptoms and radiographic changes that are similar to tuberculosis. On the other hand, MAC infection in nodular/bronchiectatic chronic lung disease is typically found in non-smoking women over the age of 50 without a preexisting history of the underlying pulmonary disease. However, bronchiectasis is invariably present at the time of MAC infection diagnosis (54).

#### 2.3.2 Immune defects

The reason for developing MAC infection in hosts with immune defects is still a topic of debate. One study found that IFN-γ, TNF-α, and IL-12 may help protect against MAC, while IL-10 could have an immunosuppressive effect on MAC (55). This is consistent with another study that showed a reduced IFN-γ response to mitogen stimulation in patients with pulmonary MAC infection (56). The dysfunction of T cells has also been demonstrated in the development of MAC infection. More recently, it has been suggested that concurrent innate and adaptive dysregulation may play a role in either the development or persistence of MAC infection. While T-cell exhaustion appears to be a significant factor contributing to MAC infection, excessive upregulation of proinflammatory pathways also enhances MAC’s persistence (57).

#### 2.3.3 Genetic predisposition

Recent data suggests that aside from environmental factors, genetic predisposition may play a significant role in the susceptibility to pulmonary NTM infection. Two key genes, the cystic fibrosis transmembrane regulator (CFTR) and the TTK protein kinase gene, have been identified as potential explanations (58–60). Specifically, genetic linkage analysis has shown a connection between chromosome 6q12-q16 and the gene *TTK*, indicating its involvement in susceptibility to pulmonary MAC infection through its role in host DNA damage repair and cell survival (59).

Mutations in the *CFTR* gene, which encodes a protein that functions as a chloride channel in epithelial cells, are responsible for cystic fibrosis and are more commonly found in Southern countries than in Asia (61). In a study of 300 Japanese pulmonary MAC infection cases, an association was found between pulmonary MAC and three *CFTR* gene variations (TG repeat, polyT, and M470V), specifically with the ISV8-T5 allele (61). Additionally, a genome-wide association study (GWAS) using whole genome data from cystic fibrosis patients discovered that the CHP2 region on chromosome 16, which is related to MAC infection, is associated with severe cystic fibrosis (62). Another GWAS on 1,066 patients with pulmonary MAC revealed a disease susceptibility SNP (rs109592) on chromosome 16, which was significantly less common in pulmonary MAC cases (odds ratio 0.54, *p* = 1.6 x 10^-13^) (63). This SNP is located in the intronic region of CHP2, which controls pH through a sodium-hydrogen ion exchanger expressed in epithelial cells (64), indicating the potential importance of airway epithelial cells in pulmonary MAC.

Familial clustering of MAC infection is another theory related to genetic predisposition. Kuwabara and colleagues (65) documented a series of cases where siblings had pulmonary MAC disease. Their Restriction Fragment Length Polymorphism (RFLP) analysis using insertion sequencing as a probe showed that the bacteria detected in sibling cases were distinct strains. This corresponds to the idea that there may be immunological impairment in the host’s immune defense mechanism. In a report from the United States, the National Institutes of Health (NIH) found 120 cases of pulmonary NTM infection in six families, where a parent and child or siblings had the disease. Five of these families had three or more cases of pulmonary MAC infection within the same family, suggesting the presence of a disease susceptibility gene (66).

Accordingly, to this evidence, it suggests that pulmonary MAC infection is likely a complex disease involving combinations of variants across gene categories, as well as environmental factors that increase susceptibility to infection. This seems to be particularly true for individuals with bronchiectasis and cystic fibrosis. Understanding this genetic overlap may be crucial for identifying the disease susceptibility genes and pathogenesis of MAC infection in the future.

### 2.4 Gut–lung axis and pulmonary non-tuberculous mycobacteria

The immune responses against NTM and/or the treatments may influence the host’s inflammatory responses, especially macrophages, and interact with other microorganisms (bacteria, fungi, and viruses) within the lung microbiome (67). For example, during active NTM infection, activated macrophages might aggressively kill bacteria, especially less virulent bacteria, thereby selectively elevating the levels of some bacteria, especially the more pathogenic ones. Indeed, Proteobacteria (mainly Gram-negative) are elevated in the lung microbiota of patients with NTM (24). Notably, virota alterations after bacterial population changes might be because of differences in bacteriophages since viruses are intracellular organisms, and changes in viruses in host cells because of changes in bacteria are less likely (68). During NTM infection, the lung microbiota is altered by host immune responses as well as by the communication between NTM and other microbes (referred to as “microbial crosstalk or microbe–microbe interaction”). Crosstalk can occur within the bacterial kingdom (bacteria–bacteria interaction) or with non-bacterial microbes (fungi and viruses), i.e., interkingdom crosstalk (69). Microbial crosstalk might be because of bacterial competition in nutrient-limited contexts or the secretion of some molecules (70). Interestingly, in addition to altering the lung microbiota, NTM may also change gut microbiota, for example through immune response activation. Moreover, through a two-way communication referred to as the “gut–lung axis”, gut microbiota changes might also affect lung NTM (69). Although NTM might be localized in specific organs, it might alter the gut microbiota because activated macrophages circulate through all organs, including the gut. Indeed, systemic macrophage depletion using liposomal clodronate alters gut microbiota (71). However, gut microbiota also affects lung NTM because some small gut microbe metabolites, such as arginine, activate lung immunity, thereby reducing NTM burden (72). Indeed, the translocation of gut microbiota-produced small molecules and/or small molecules from the digestion of gut contents, is an underlying mechanism of the crosstalk between the lungs and other organs (73). Thus, administering arginine or arginine production-enhancing probiotics might be beneficial for NTM treatment (72). Hence, a better understanding of the gut–lung axis might improve our understanding of NTM pathogenesis and identify new therapeutic modalities.

### 2.5 Experimental animal models for MAC infection

Animal models play a crucial role in studying the pathogenesis of MAC infection and in testing new therapies. Commonly used models include mice, zebrafish, rabbits, and non-human primates. Recent studies involving mouse models have offered valuable insights into MAC infection pathogenesis, immune responses, and potential therapeutic strategies.

Mice are particularly useful for examining the immune response to MAC infection, especially in immunocompromised hosts that mimic human conditions, such as HIV/AIDS or other states of immunosuppression. MAC infection in mice typically affects the lungs, liver, and spleen, with granuloma formation being a significant feature of the host immune response (74). Research has shown that T-cell-mediated immunity, especially through Th1 responses (involving cytokines like IFN-γ and TNF-α), is essential for controlling MAC infections. Mice deficient in IFN-γ or TNF-α are more susceptible to widespread MAC disease. Interestingly, macrophages have a dual role in both controlling and disseminating MAC infection, as the mycobacteria can survive and replicate within these cells (74, 75).

In terms of genetic susceptibility, certain mouse strains, such as C57BL/6 and BALB/c, have been utilized to study the genetic factors influencing MAC infection. For instance, C57BL/6 mice are more resistant to MAC due to their robust Th1 responses, whereas BALB/c mice are more susceptible (76, 77). Additionally, polymorphisms in the Nramp1 (Slc11a1) gene have been shown to influence susceptibility to MAC in mice, similar to their effects in humans (76, 77).

Chronic MAC infection models in mice have been developed to investigate long-term immune responses and the emergence of drug resistance (78). These models indicate that persistent infection leads to chronic inflammation and tissue damage, reflecting the situation seen in human cases (79). Recent studies have also highlighted the use of mouse models to test the effectiveness of various antibiotics, such as clarithromycin, azithromycin, and ethambutol, as well as immunomodulatory agents like IFN-γ against MAC (80). Combination therapy has proven to be more effective than using a single agent, as it reduces bacterial load and prevents relapse. Moreover, host-directed therapies (HDTs) targeting immune pathways (e.g., autophagy, cytokine modulation) are being explored as adjuncts to antibiotics (81).

While mouse models have significantly contributed to our understanding of MAC infection pathogenesis, immune responses, and therapeutic strategies, there are notable limitations (82). One major issue is the difference in immune responses between mice and humans, which must be considered when applying these findings to clinical settings (82). Zebrafish are becoming increasingly popular for studying MAC infection due to their transparent embryos, which allow for real-time imaging of host-pathogen interactions (83). Additionally, rabbits develop granulomatous lesions that resemble those seen in humans, making them valuable for researching pulmonary MAC disease (84). Primates also closely mimic human MAC disease, but their use is less common due to ethical concerns and high costs (85).

## 3 Clinical manifestations of non-tuberculous mycobacteria infections

**Figure 3:**
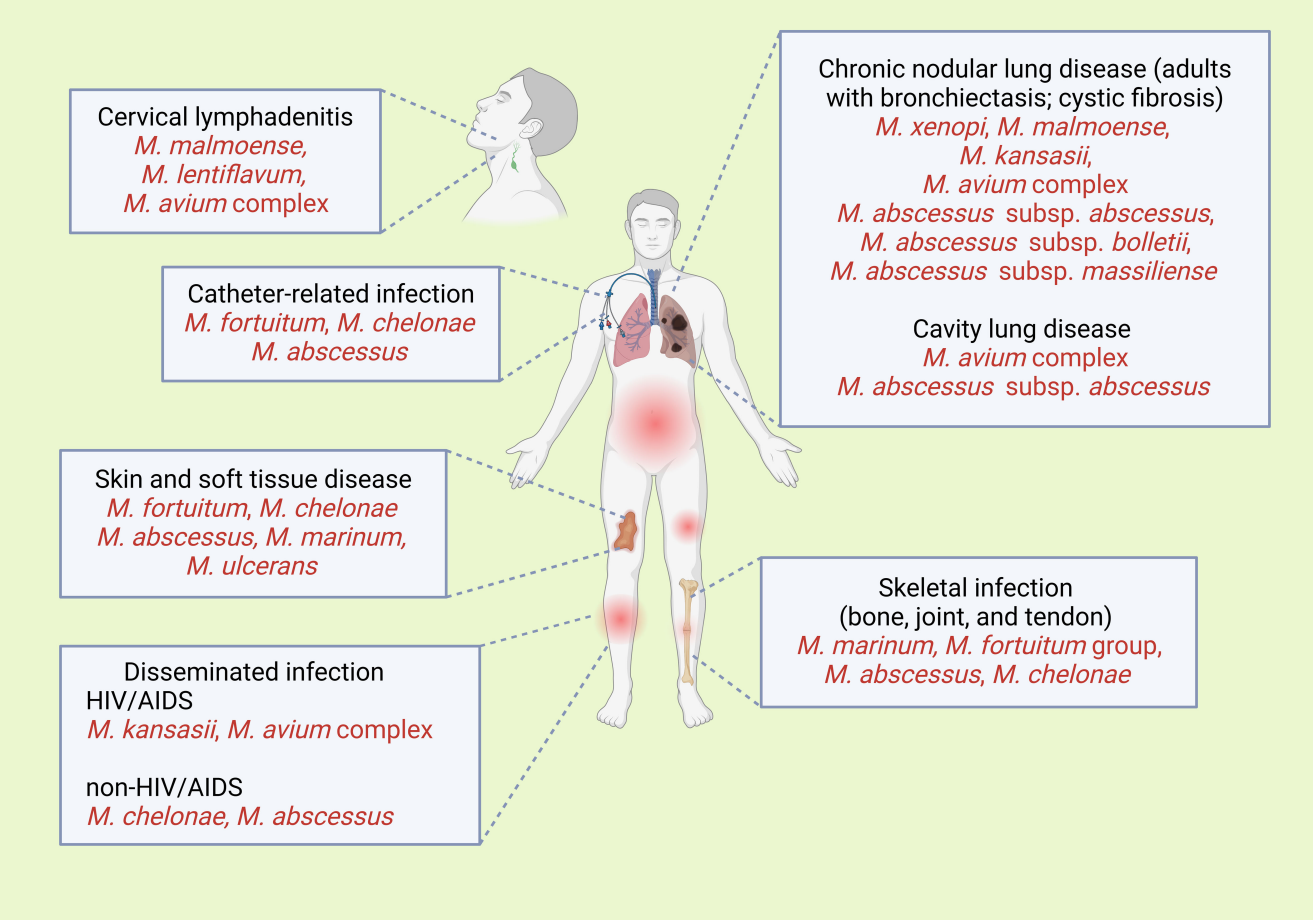
Clinical signs of the common presentations of infections with various non-tuberculosis mycobacterial species. Created with BioRender.com.

NTM infections have a diverse range of clinical manifestations (9, 12), which depend on the transmission route (**Figure 3**). Most cases are characterized by an insidious onset, are asymptomatic, and tend to follow a chronic course. Diagnosis is based on a combination of radiologic, pathologic, and microbiological examinations, including culture and isolation methods, or genomic analyses to confirm bacterial presence. Of note, MAC is typically found primarily in pulmonary MAC infection, followed by disseminated in HIV/AIDS, cutaneous, and lymphadenitis.

### 3.1 Diagnostic criteria for non-tuberculous mycobacteria infections

The distinction between colonization and disease in the context of NTM is critical for appropriate clinical management. NTM colonization refers to the isolation of NTM from respiratory specimens (e.g., sputum or bronchoalveolar lavage) in the absence of clinical symptoms, radiographic changes, or histopathologic evidence of tissue invasion, often representing environmental contamination or transient presence in individuals with underlying lung conditions such as COPD or bronchiectasis. In contrast, NTM disease is diagnosed when there is evidence of tissue invasion, clinical symptoms (e.g., chronic cough, sputum production, weight loss, or fatigue), and radiographic abnormalities (e.g., nodules, cavitation, or multifocal bronchiectasis), supported by microbiological criteria such as positive cultures from at least two separate sputum samples or one bronchial wash/lavage, or histopathologic findings of granulomatous inflammation or acid-fast bacilli on biopsy. The American Thoracic Society/Infectious Diseases Society of America (ATS/IDSA) guidelines emphasize the importance of integrating clinical, radiographic, and microbiological data to differentiate colonization from disease, particularly in high-risk populations such as those with structural lung disease or immunosuppression, as NTM species like *Mycobacterium avium complex* and *M. abscessus* are more likely to cause disease (5, 86).

**Figure 4:**
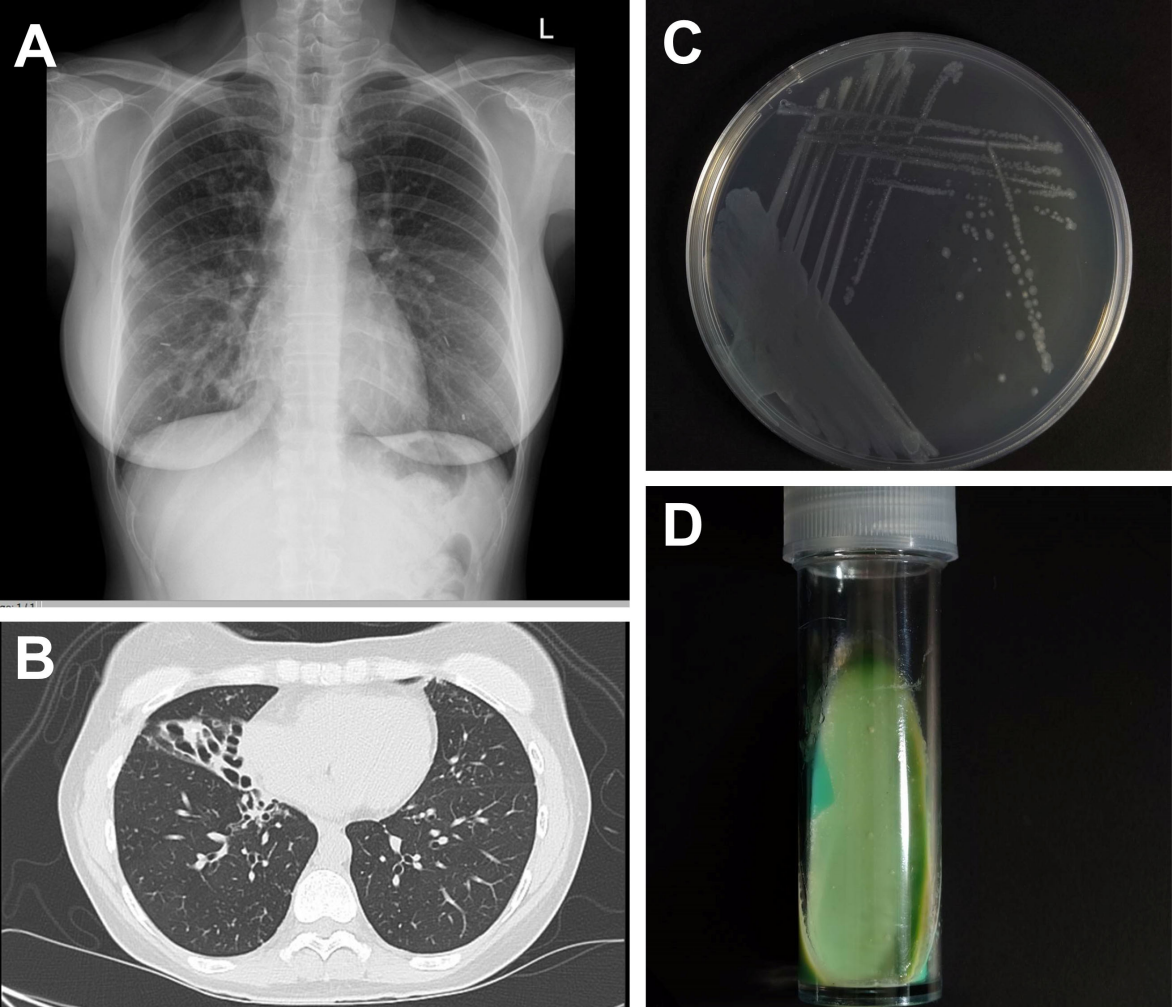
Investigations of *Mycobacterium avium* complex. **(A)** Chest radiography shows interstitial infiltration at right middle lung field and **(B)** computed tomography demonstrates centrilobular with tree-in-bud appearance and multiple pulmonary nodules along with cylindrical bronchiectasis. The growing colonies of *M. avium* subspecies avium ATCC 25921 on **(C)** Middlebrook 7H10 medium and **(D)** Löwenstein–Jensen medium that grew at 35 C for 3 weeks.

Various societies have developed criteria for NTM diagnosis, including the American Thoracic Society (ATS), the European Society of Clinical Microbiology and Infectious Disease (ESCMID), the European Respiratory Society (ERS), and the Infectious Diseases Society of America (IDSA). The main aim of these criteria, which were developed in 2007 (5) and were still in use in 2020 (86), is to exclude environmental-derived NTM, particularly when using non-sterile testing techniques. NTM diagnosis requires that the following criteria are met and other possible diseases are excluded (5, 86):

1. NTM-compatible symptoms, such as respiratory tract symptoms, unexplained weight loss, and fever.
2. Radiology results that match the presence of nodular or cavity opacities based on chest radiography, or the presence of bronchiectasis and multiple small nodules based on a chest computed tomography scan (**Figures 4A, B**).
3. At least two NTM samples demonstrating the presence of the same NTM pathogen based on mycobacterial culture, or at least one bronchial alveolar fluid.

Additionally, the presence of granulomatous inflammation or acid-fast bacilli staining based on lung tissue pathology, and the presence of lung tissue or bronchial alveolar fluid NTM can help diagnose NTM.

Unlike pulmonary NTM, diagnosing aesthetic procedure-associated NTM requires a history of a dermatological procedure within three months before skin lesion appearance and the following criteria to be met:

1. No skin and soft tissue infection (SSTI) bacteria isolation from at least two biopsies at different times.
2. No antibiotic response within two weeks.
3. Presence of SSTI signs, including induration, furuncle, cutaneous abscess, and a deep draining tract.
4. Presence of immunosuppression risk factors, such as diabetes mellitus, transplantation, or being on immunosuppressive drugs (87).

Moreover, cutaneous MAC infection should be considered in patients with cellulitis who do not respond to antibiotics, especially in individuals with persistent nodules and ulcers, and in immunosuppressed patients showing signs of widespread MAC infection.

MAC initially consisted of two species, *M. avium* and *M. intracellulare*. Over time, other terms were used to refer to these species, such as MAI (*M. avium–intracellulare*), or to encompass other related mycobacteria, like MAIS (*M. avium–intracellulare–scrofulaceum*) and MAIX (*M. avium–M. intracellulare cluster X*, referring to unnamed mycobacteria not belonging to any of the former taxa) (88). However, before the genetic-based taxonomy of the 1990s, using only cultural and biochemical tests was not enough to differentiate the MAC members accurately. Therefore, the identification of MAC organisms in publications from those years is questionable. Based on phenotypic and genotypic tests confirming the close relatedness of different MAC organisms, the MAC has been shown to contain a wide variety of environmental and animal-associated organisms with varying degrees of pathogenicity, host preference, and environmental distribution. As a result, the complex now includes nine species of slow-growing mycobacteria, including *M. avium* (Subsp. *avium* [MAA], Subsp. *hominissuis* [MAH], Subsp. *paratuberculosis* [MAP], and Subsp*. silvaticum* [MAS]), *M. intracellulare*, *M. colombiense*, *M. chimaera*, *M. marseillense*, *M. timonense*, *M. bouchedurhonense*, *M. vulneris*, *M. arosiense*, *M. indicus pranii*, *M. yongonense*, and, a further subset of isolates of undetermined classification known as “MAC-others” as well as it should be noted that *M. scrofulaceum*, which belongs to the MAIS (*M. avium*-*intracellulare*- *scrofulaceum*) complex, is no longer grouped in MAC (89).

The identification of mycobacterial species is crucial for NTM treatment. Unfortunately, most commercial tests nowadays cannot identify the species within the complex. Because of limited NTM identification resources, it is essential that physicians comprehensively understand the fundamental aspects of laboratory tests and their treatment limitations. Microscopic examination after staining and culture using specific media are the cornerstones of the identification of mycobacteria but is relatively insensitive. In general, the gold standard for NTM isolation and diagnosis is culture in solid media, such as Middlebrook 7H10 agar (**Figure 4C**) or Löwenstein–Jensen agar (**Figure 4D**), or liquid media, such as using the BD BACTEC MGIT 960 system (5, 90, 91). However, for optimal growth, various subspecies require unique temperatures. For instance, *Mycobacterium haemophilum* and *Mycobacterium marinum* require 28–30°C, whereas *Mycobacterium xenopi* requires 42°C. On the contrary, MAC grows well in both 7H10 agar and MGIT broth. It does not show clustering or cording in broth. On agar, it typically produces small, flat, translucent, smooth colonies that occasionally exhibit a pale-yellow color. These colony morphologies differ from *M. tuberculosis*, which typically shows cording in broth and appear as rough, buff-colored colonies on agar. Moreover, the molecular technique for MAC diagnosis uses nucleic acid testing (Gen-Probe AccuProbes), which can identify MAC within 24-48 hours from culture, showing approximately 79% accuracy (92).

As recommended by the Clinical and Laboratory Standards Institutes (CLSI) M24S guidelines (93–95), broth microdilution is the antimicrobial susceptibility testing (AST) gold standard. Sensitire RAPMYCOI and SLOMYCOI plates (Thermo Fischer Scientific, Cleveland, OH, USA) are used for RGM and slow-growing mycobacteria (SGM), respectively (96, 97). However, because standard methods require high levels of expertise, several molecular techniques have replaced these gold standards. They include the Anyplex MTB/NTM real-time detection system (Seegene, Seoul, South Korea) (98), which uses real-time polymerase chain reaction and the INNO-LiPA Mycobacteria v2 (Fujirebio, Tokyo, Japan) (99) and the Genotype Mycobacteria CM, AS, NTM-DR (Hain Lifescience, Nehren, Germany) systems (100–102), which use PCR and reverse DNA hybridization methods (**Table 1**).

**Table 1:** Summary of NTM test properties and species identification performance (91, 96–102).

	Genotypic methods			Phenotypic methods
	Anyplex MTB/NTM real-time detection (Seegene, Seoul, South Korea)	INNO-LiPA Mycobacteria v2 (Fujirebio, Tokyo, Japan)	Genotype Mycobacteria CM, AS, NTM-DR (Hain Lifescience, Nehren, Germany)	Solid and liquid media
Duration	2–3 days	3–7 days	3–7 days	2–8 weeks
Sensitivity	++	+	+	++++
Identification methods	16S rRNA gene	16S–23S rRNA spacer region	23S rRNA gene	N/A
*Mycobacterium* spp.	+	+	+	
*M. tuberculosis* complex	+	+	+	
Rapid growing mycobacteria	N/A			
*M. abscessus* complex		+	+ ^CM, NTM-DR^	
susp. *abscessus*		–	+ ^NTM-DR^	
susp. *bolletii*		–	+ ^NTM-DR^	
susp. *massillense*		–	+ ^NTM-DR^	
*M. chelonae* complex		+	+ ^CM, NTM-DR^	
*M. fortuitum* complex		+	+ ^CM^	
Slow growing mycobacteria				
*M. avium* complex		+	+ ^CM, NTM-DR^	
*M. avium*		+	+ ^CM, NTM-DR^	
*M. intracellulare*		+	+ ^CM, NTM-DR^	
*M. chimaera*		–	+ ^NTM-DR^	
*M. genavense*		+	+^AS^	
*M. gordonae*		+	+ ^CM^	
*M. haemophilum*		+	+ ^AS^	
*M. kansasii*		+	+ ^CM, AS^	
*M. malmoense*		+	+ ^CM^	
*M. marinum*		+	+ ^CM^	
*M. scrofulaceum*		+	+ ^CM^	
*M. simiae*		+	+ ^AS^	
*M. szulgai*		–	+ ^CM, AS^	
*M. terrae* complex		–	–	
*M. ulcerans*		+	+ ^CM, AS^	
*M. xenopi*		+	+ ^CM^	
Other NTM	N/A	*M. celatum*	*M. asiaticum* ^AS^	
		*M. smegmatis*	*M. celatum* ^AS^	
			*M. gastri* ^AS^	
			*M. goodii* ^AS^	
			*M. heckeshornense* ^AS^	
			*M. interjectum* ^CM^	
			*M. intermedium* ^AS^	
			*M. lentiflavum* ^AS^	
			*M. mucogenicum* ^AS^	
			*M. phlei* ^AS^	
			*M. shimoidei* ^AS^	
			*M. smegmatis* ^AS^	
Antimycobacterial agent sensitivity tests	Unable	Unable	Detection of *erm*(41) and *rrl* mutations for macrolides and aminoglycosides resistance, respectively, (NTM-DR, Hain Lifescience)	Minimum inhibitory concentration (MIC)

+refers to the sensitivity of testing from sputum.

AST is crucial for the management of MAC infections. MAC testing is typically done using antimycobacterial agents like macrolides, with clarithromycin being preferred over azithromycin because it dissolves at high concentrations. However, the minimum inhibitory concentration (MIC) of other drugs, including ethambutol, rifampicin, and rifabutin, do not correlate with clinical outcomes (95, 103). The value of expanded *in vitro* susceptibility testing for macrolide-resistant MAC isolates has yet to be demonstrated. Acquired MAC resistance to macrolides and amikacin is caused by 23S rRNA (*rrl*) and 16S rRNA (*rrs*) mutations, respectively (104, 105). Therefore, if the clinical condition does not improve and persistent MAC is detected after treatment, AST should be repeated. In cases of disseminated and chronic lung disease, repeat AST is recommended three and six months after treatment, respectively (5).

Currently, broth microdilution based on the American Type Culture Collection strain and CLSI M24S protocols is the recommended AST method. Sensitire RAPMYCOI and SLOMYCOI (Thermo Fischer Scientific, Cleveland, OH, USA), which are used for RGM and SGM, respectively (96, 97), use different antimycobacterial agents and dosages (**Table 2**). These tests are critical for the identification of the most effective treatment option and ensuring successful patient outcomes.

**Table 2:** Recommended antimycobacterial agent concentrations for use in AST (93, 95–97).

Antimycobacterial agent	Recommended antimycobacterial agent concentration (μg/ml)			
	RAPMYCOI	RAPMYCO2	SLOWMYCOI	SLOWMYCO2
Amikacin	1-64	1-256	1-64	1-256
Amoxicillin/clavulanic acid (2:1)	2-1-64/32	–	–	–
Cefepime	1-32	–	–	–
Cefoxitin	4-128	1-128	–	–
Ceftriaxone	4-64	–	–	–
Ciprofloxacin	0.12-4	0.12-4	0.12-16	0.12-8
Clarithromycin	0.06-16	0.06-16	0.06-64	0.06-64
Clofazimine	–	0.03-4	–	0.015-4
Doxycycline	0.12-16	0.12-8	0.12-16	0.12-8
Imipenem	2-64	0.008-32	–	–
Linezolid	1-32	1-32	1-64	1-32
Minocycline	1-8	–	–	0.06-8
Moxifloxacin	0.25-8	0.015-4	0.12-8	0.015-4
Tigecycline	0.015-4	0.03-2	–	
Tobramycin	1-16	0.12-16	–	–
TMP/SMX	0.25-8	0.25-4	0.12-8	0.25-4
Isoniazid	–	–	0.25-8	–
Rifampin	–	–	0.12-8	0.004-4
Rifabutin	–	–	0.25-8	0.12-4
Ethambutol	–	–	0.5-16	–
Ethionamide	–	–	0.3-20	–
Streptomycin	–	–	0.5-64	0.5-32

TMP/SMX, Trimethoprim.

In general, *M. avium* complex AST, identification for susceptibility tests requires careful interpretation. Although it is recommended that test results be interpreted on day 7 of testing, interpretation should be delayed until day 10 or 14 of testing in case growth is insufficient. On the other hand, for insidious NTM, such as *Mycobacterium marinum* and *Mycobacterium Kansassii*, interpretation should be done after 7–14 days, whereas for *Mycobacterium xenopi* and *Mycobacterium malmoense*, 3–4 weeks are required. RGM results should be interpreted 18 hours after testing and cases without growth in the positive control should be given more inoculation time and interpreted on days 3–5. The test must be repeated if there is no growth. Moreover, to detect inducible macrolide resistance, the interpretation of clarithromycin’s MIC should be done on day 14. Additionally, in RGM cases that lack the *erm* gene or have a non-functional *erm* gene, such as *M. abscessus* subsp. *massiliense*, *M. chelonae*, *Mycobacteroides immunogenum*, the *Mycobacterium mucogenicum* group, *Mycobacterium peregrinum*, and *Mycobacterium senegalense*, clarithromycin susceptibility can be interpreted on days 3–5 of the test without additional interpretation on day 14.

The occurrence of either contamination or false-positive NTM cultures represents a critical diagnostic challenge, particularly in patients with extended hospitalization periods. Prolonged hospital stays increase the risk of environmental exposure to NTM, commonly found in hospital water systems, medical devices, and disinfectants. These organisms can contaminate clinical specimens during collection, handling, or processing, leading to misleading positive culture results. False-positive NTM cultures can also arise from laboratory errors, such as cross-contamination between samples or misinterpretation of culture findings, especially when NTM are present as colonizers rather than true pathogens (5, 106). This issue is particularly problematic in immunocompromised patients or those with underlying lung diseases, where distinguishing between contamination, colonization, and actual infection is crucial for appropriate clinical management. Misinterpretation of NTM culture results can lead to unnecessary antimicrobial therapy, which is often prolonged and associated with significant side effects and increased healthcare costs. Therefore, clinicians and microbiologists must exercise caution in interpreting NTM cultures, particularly in the context of prolonged hospitalization, and consider corroborative evidence such as clinical symptoms, radiographic findings, and repeat sampling to confirm the diagnosis (5, 106).

Because AST is crucial in determining antimycobacterial drug effectiveness, AST results interpretation and reporting are of paramount significance. The CLSI M24S sets the breakpoint values for various NTM, including MAC, *Mycobacterium kansasii*, and other SGM. MIC results should be reported in MIC units (μg/ml) and susceptibility tests should be reported using the S (susceptible), I (intermediate), and R (resistant) categories (95) (**Table 3**). For some medications, only MICs should be reported, and not breakpoint values. These guidelines ensure consistent and accurate reporting of AST results and guide informed clinical decisions about antimycobacterial agent use.

**Table 3:** Breakpoint recommendations in terms of SGM and RGM based on CLSI M24S 2023.

Mycobacterial group	Antimycobacterial agent	Minimal inhibitory concentration (μg/ml)		
		Susceptible	Intermediate	Resistant
Rapid growing mycobacteria	Amikacin	≤16	32	≥64
	Cefoxitin	≤16	32–64	≥128
	Ciprofloxacin^a^	≤1	2	≥4
	Clarithromycin^b^	≤2	4	≥8
	Doxycycline^c^	≤1	2–4	≥8
	Imipenem	≤4	8–16	≥32
	Linezolid	≤8	16	≥32
	Meropenem	≤4	8–16	≥32
	Moxifloxacin	≤1	2	≥4
	Tobramycin	≤2	4	≥8
	TMP/SMX	≤2/38		≥4/76
Slow growing mycobacteria				
*M. avium* complex	First-line			
	Amikacin (intravenous)	≤64	32	≥64
	Amikacin (liposomal, inhaled)	≤64		≥128
	Clarithromycin^a^	≤8	16	≥32
	Second-line			
	Linezolid^d^	≤8	16	≥32
	Moxifloxacin^d^	≤1	2	≥4
*M. kansasii*	First-line			
	Clarithromycin^b^	≤8	16	≥32
	Rifampin	≤1		≥2
	Second-line			
	Amikacin (intravenous)	≤16	32	≥64
	Ciprofloxacin	≤1	2	≥4
	Doxycycline/ minocycline	≤1	2–4	≥8
	Linezolid	≤8	16	≥32
	Moxifloxacin	≤1	2	≥4
	Rifabutin	≤2		≥4
	TMP/SMX	≤2/38		≥4/76

a Equivalent to the levofloxacin susceptibility test.

b Equivalent to other in macrolides (azithromycin, roxithromycin) susceptibility tests.

c Equivalent to the minocycline susceptibility test.

d No *in vivo* data on the efficacy of *M. avium* complex treatment.

TMP/SMX, trimethoprim/sulfamethoxazole.

Regarding wild-type MAC, it is critical to conduct a macrolide susceptibility test because of potential resistance development within 2–3 months after monotherapy or combination treatments, particularly at the 23S rRNA V-domain. If the test results are intermediate or indicate resistance, a confirmation test and species identification should be performed.

CLSI recommends repeating RGM susceptibility tests, especially if the result does not indicate resistance (95). For instance, if amikacin’s MIC against the *M. abscessus* complex is ≥64 μg/ml, a confirmation test must be done. Furthermore, the *M. fortuitum*, *Mycobacterium mucogenicum*, and *Mycobacterium smegmatis* groups should demonstrate imipenem susceptibility and the test must be repeated if the MIC exceeds 8 μg/ml and the results must be reported within three days. Moreover, imipenem susceptibility cannot be generalized to ertapenem and meropenem even if they belong to the same class (95). Similarly, susceptibility to tobramycin, which is often used for *M. chelonae* treatment, must be tested if the MIC is >4 μg/ml and the test should be repeated along with species identification based on *rpoB* testing. In summary, a comprehensive understanding of the timing and interpretation of susceptibility testing is fundamental for accurate mycobacterial diagnosis and effective treatment. For informed decisions, the clinicians must gather all pertinent data and interpret them based on clinical signs.

### 3.2 Non-tuberculous mycobacteria infection treatment challenges

Treatment of NTM infections is a long-term process that warrants the consideration of potential side effects. Additionally, other factors that affect treatment must be considered (17, 107) (**Table 4**). However, the long-term clinical effects of the treatment are unknown, and recent studies have not established a correlation between early treatment and a favorable prognosis (108, 109). Thus, for patients who exhibit mild symptoms or intermittent presentation with subtle radiographic changes, it is reasonable to defer treatment. The need to balance disease progression with treatment-related toxicity and drug resistance development must be considered carefully. Importantly, decision-making should be shared with patients and their families, and patients should be informed about the treatment’s risks, including its success rates and adverse reactions. Ultimately, the decision on whether to proceed with the treatment lies with the patient and their family.

**Table 4:** Factors to be considered during shared decision-making before treatment and on withholding antimycobacterial treatments.

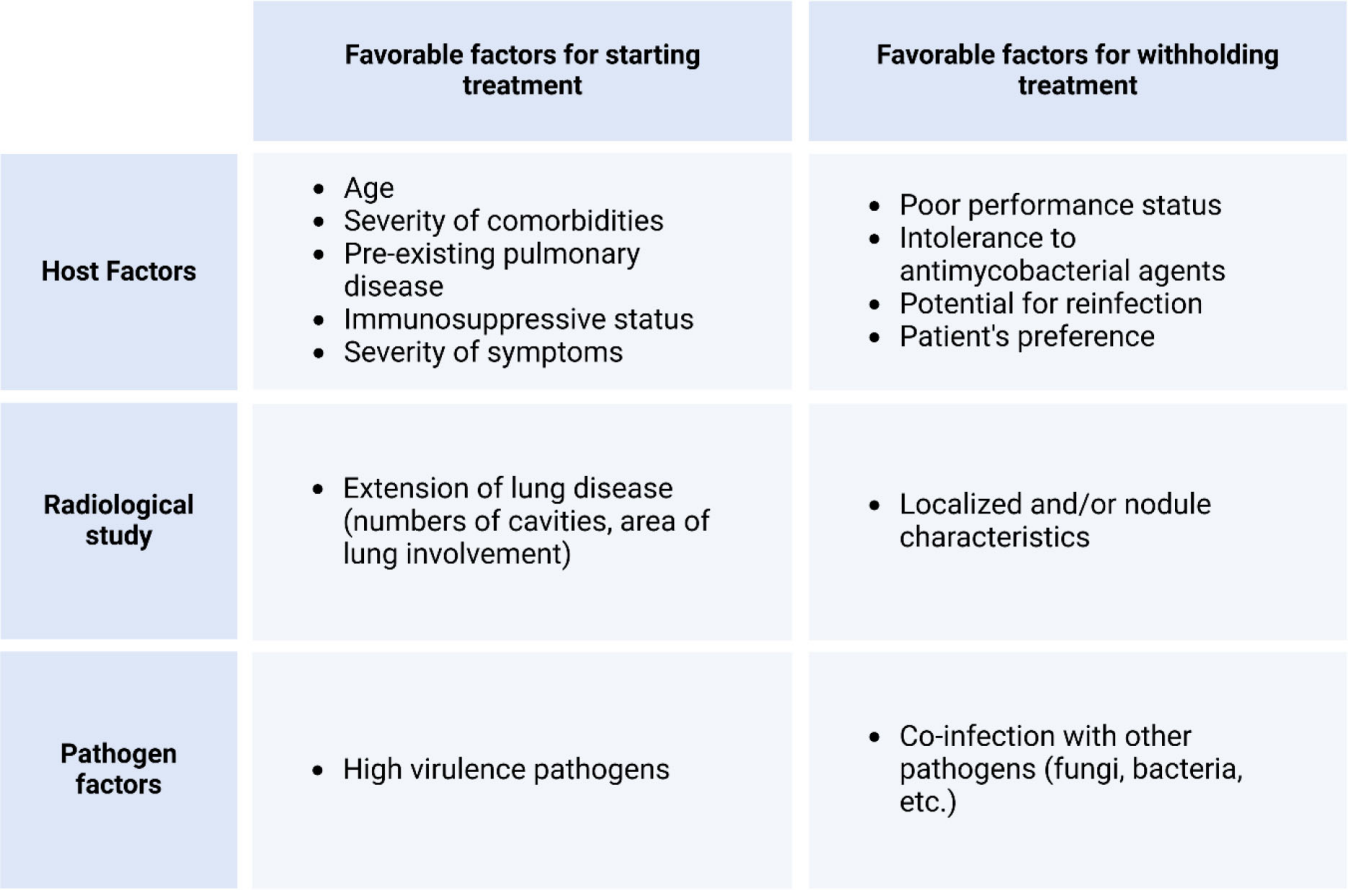

Unless treatment initiation benefits outweigh the risks of treatment withholding, treatments of NTM infection typically involve a period of watchful waiting. For instance, treatment may be necessary if a sputum smear reveals acid-fast bacilli or if cavity lung disease is detected (86), owing to the cavity disease is destructive and associated with more rapid progression. Regarding smear-negative nodular bronchiectatic disease, the decision to treat or choose observation depends on the patient’s clinical presentation and status. Observation is suitable for those with mild MAC or other medical issues that outweigh the morbidity from MAC infection. However, the development of a new cavity or worsening nodularity are signs that treatment should be initiated since the disease is progressing. Other treatment-necessitating factors include disease progression, preexisting lung diseases, immune system status, and the presence of high-virulence pathogenic strains. These factors represent the three aspects of disease progression, i.e., the host, pathogen, and transmission (or radiographic phenotype) (**Table 4**).

Optimal treatments of NTM infection require a combination of non-pharmacological and pharmacologic interventions, or at the very least, a preliminary non-pharmacological approach to carefully monitor its effects before initiating pharmacotherapy. The ATS and ERS recommend a treatment strategy that involves a holistic intervention underscored by thorough patient evaluation and individualized therapies, including exercise regimens, patient education, and behavioral changes aiming to improve physical and mental health, promote long-term patient compliance, and encourage the adoption of health-promoting behavior (110).

### 3.3 Principal *Mycobacterium avium* complex management

**Figure 5:**
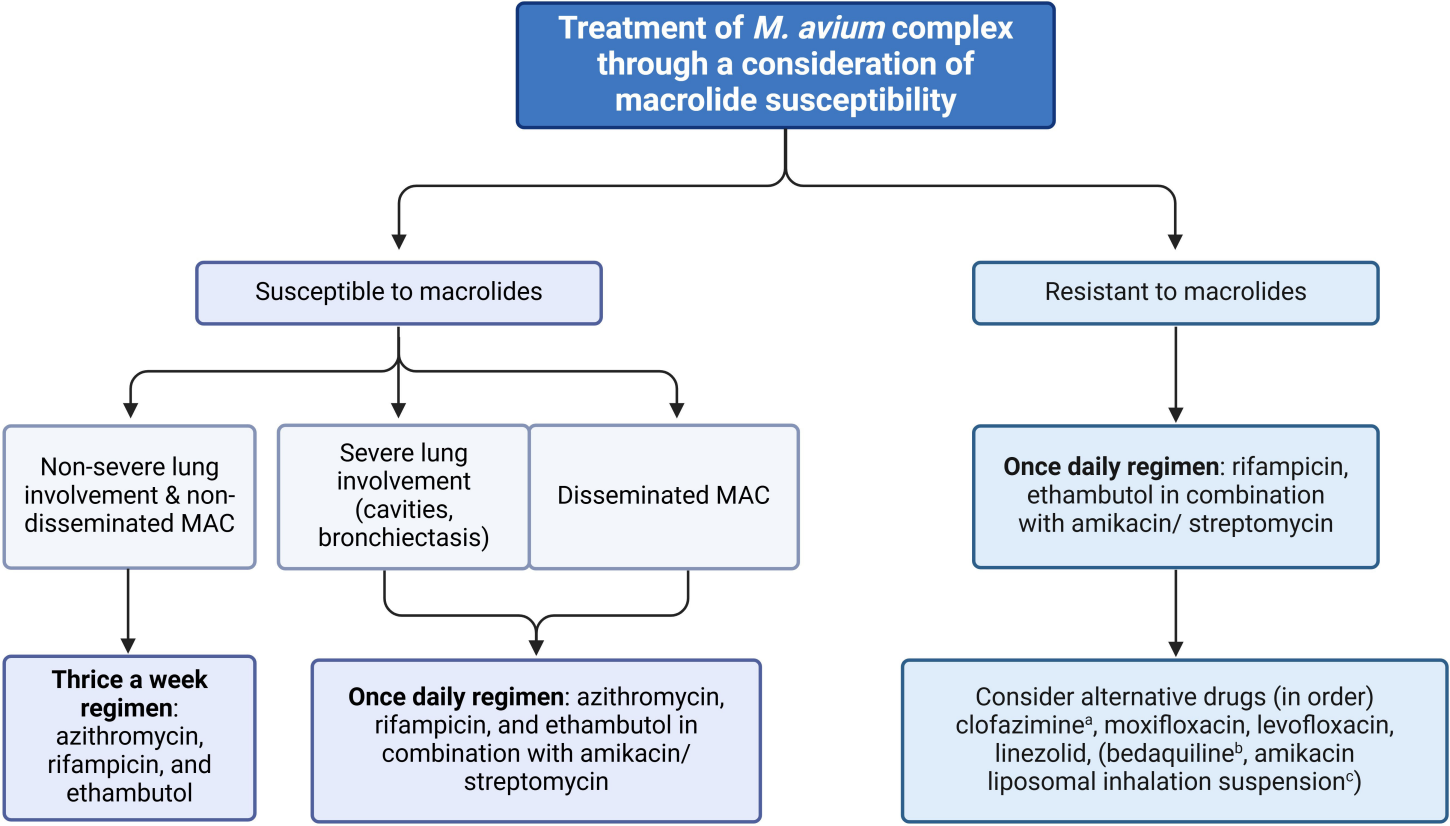
Flowchart of treatment for *M. avium* complex (MAC) steps (26, 49). MAB: *M. abscessus* subsp. *Abscessus*, MBO: *M. abscessus* subsp. *Bolletii*, MMA: *M. abscessus* subsp. *Massillense.* ^a^Recommended for use as the first-line drug as still susceptible over 90% along with additive effects with amikacin. ^b^This drug is preserved only for cases that lack alternative drugs and it cannot be combined with rifampicin. ^c^This treatment is exclusively reserved for cases in which pneumonia does not respond to any other alternative medication.

The primary approach to managing MAC infection involves using three types of antibacterial regimens that are effective against the specific sensitivities of the pathogen, instead of using two or fewer drugs, unless patients are intolerant to three drug regimens. As an alternative, a two-drug regimen, such as a combination of macrolide and ethambutol, is considered reasonable. Macrolides, which have exhibited a high treatment success rate when combined with ethambutol and drugs of the rifamycin group, such as rifabutin or rifampicin, are the most crucial medications for MAC infection treatment (111, 112). Ethambutol is essential for increasing pathogen elimination efficacy and preventing macrolide resistance. This drug combination was recommended as the first-line regimen by the ATS/ERS/ESCMID/IDSA association in 2020 (86) and the BTS association in 2017 (106). In cases of severe or disseminated infection, aminoglycoside co-administration is advisable, and it is essential to test for macrolide and aminoglycoside susceptibility before treatment. Typically, MAC infection treatment plans depend on disease severity and AST results (**Figure 5**).

Macrolides, ethambutol, and rifamycin are important groups of antimicrobial drugs and are often used to treat bacterial infections (15, 86, 106, 113). Macrolides should always be considered in cases with antimicrobial drug sensitivity. Azithromycin is particularly useful because it has lower interaction with rifampicin, lower pill burden, fewer adverse reactions, and can be administered once daily. However, studies indicate that in patients with disseminated MAC infection, clarithromycin is more effective than azithromycin in clearing circulating pathogens (114). Of note, the treatment of disseminated MAC infection in patients with AIDS is complicated by the drug interactions between rifampicin, a cornerstone of MAC infection therapy, and antiretroviral (ARV) agents. Rifampicin is a potent inducer of cytochrome P450 (CYP) enzymes, particularly CYP3A4, which metabolizes many ARVs, leading to reduced plasma concentrations and potential treatment failure (115). For example, rifampicin significantly decreases the levels of protease inhibitors (PIs) such as lopinavir, atazanavir, and darunavir, which are substrates of CYP3A4. Coadministration of rifampicin with these PIs can reduce their area under the curve (AUC) by up to 75-95%, necessitating dose adjustments or alternative regimens (116). Similarly, non-nucleoside reverse transcriptase inhibitors (NNRTIs) like efavirenz and nevirapine are also affected, though efavirenz may be used with rifampicin with a dose increase to 800 mg daily to counteract the induction effect (117). In contrast, rifampicin has a lesser impact on integrase strand transfer inhibitors (INSTIs) such as dolutegravir and raltegravir, making them preferable in rifampicin-containing regimens, though dolutegravir may require twice-daily dosing to maintain therapeutic levels (118).

To mitigate these interactions, rifabutin, a rifamycin with weaker CYP3A4 induction, is often substituted for rifampicin. Rifabutin has fewer interactions with ARVs, though dose adjustments are still required when used with PIs. For instance, rifabutin doses must be reduced to 150 mg every other day when coadministered with ritonavir-boosted PIs to avoid toxicity (119). Despite these challenges, the concurrent management of MAC infection and HIV infection requires careful selection of ARVs, therapeutic drug monitoring, and close clinical follow-up to ensure both infections are adequately controlled while minimizing adverse effects and drug interactions.

For non-severe lung infections that do not involve cavities or emphysematous characteristics, a thrice-weekly treatment regimen can be administered in combination with ethambutol and rifamycin (rifampicin or rifabutin), for example, azithromycin (500 mg three times per week) PLUS rifampicin (600 mg three times per week) PLUS ethambutol (25 mg/kg three times per week). However, a once-daily regimen is recommended for cases with severe lung infection or disseminated infection (86, 113), for example, azithromycin (250 to 500 mg daily) PLUS rifampicin (600 mg daily) PLUS ethambutol (15 mg/kg daily).

In the case of mild severity of MAC infection, it is recommended to start treatment with a low initial dose while closely monitoring for any adverse reactions to prevent non-adherence. It’s worth noting that if azithromycin is intolerable, clarithromycin (1000 mg three times per week) can be used as an alternative. A lower dose of clarithromycin (500 mg once daily) should be considered in patients with a reduced renal function. Similarly, for individuals experiencing rifampicin-related hepatotoxicity, rifabutin (300 mg three times per week) is suggested. Although rifabutin demonstrates greater *in vitro* activity against MAC than rifampicin, it is uncertain if this holds true in clinical *in vivo*. In addition, parenteral aminoglycosides (amikacin or streptomycin, 10-15 mg/kg three times per week) can also be considered for severe lung infections or disseminated infections. They are typically used for 2–4 weeks in combination with other major drug groups and can be administered once daily or thrice weekly via injection. Aminoglycosides are also used in macrolide-resistant cases (15, 86, 106, 113). In persons with reduced kidney function, which is defined as having an estimated glomerular filtration rate of less than 60 mL/min/1.73 m^2^, recommending caution use of aminoglycosides. As an alternative, it is suggested to use amikacin liposome inhalation suspension at dosing of 590 mg once daily. However, in situations where resources are limited, inhaled parenteral amikacin can be used as a potential alternative. The typical dosage for this is 250 to 500 mg per day, to be administered three to seven days per week for the entire duration of treatment.

If one of the first-line drugs (macrolides, ethambutol, or rifamycin) is unavailable, three alternative drugs can be considered. The first choice, clofazimine, has a sensitivity of >90% *in vitro* and exhibits an additive effect when combined with amikacin (86, 113, 120, 121). Subsequently, moxifloxacin, levofloxacin, and linezolid can be considered in that order. However, their clinical outcomes remain controversial although they exhibit high sensitivity *in vitro* (86, 111, 113). Additionally, quinolone or linezolid have minimal activity, and certain combinations (i.e., quinolone plus macrolide) do not prevent macrolide resistance. Although bedaquiline is also an option, it should only be used when alternative drugs are unavailable because of limited clinical data and it should not be combined with rifampicin (113, 120, 122). Notably, the interaction between bedaquiline and rifampicin can be complex due to rifampicin’s potent induction of cytochrome P450 (CYP) enzymes, particularly CYP3A4. Bedaquiline is metabolized primarily by CYP3A4, and when co-administered with rifampicin, the induced metabolism can significantly reduce bedaquiline’s plasma concentrations, potentially compromising its efficacy (123). Studies have shown that rifampicin can decrease bedaquiline exposure by up to 50%, necessitating careful consideration of dosing strategies or alternative regimens to avoid subtherapeutic levels of bedaquiline (124). This interaction underscores the importance of monitoring and potentially adjusting treatment protocols when these drugs are used together in MAC infection therapy.

An intermittent regimen is recommended for the initial treatment of non-cavitary bronchiectatic disease. However, if patients have severe MAC infection or cavitary disease, or have not responded to previous regimens, intermittent medication dosing may not be sufficient. In such cases, it is recommended to switch to a daily regimen if sputum cultures remain positive after six months of intermittent treatment (125). Treatment failure is considered for such patients and adding amikacin liposomal inhalation suspension (ALIS) is recommended (86). However, some patients may show clinical and radiographic improvement but still have persistent positive sputum culture for MAC. In such cases, continuing with a macrolide-based treatment plan along with the addition of ALIS 590 mg once daily for the entire duration of MAC infection treatment and switching to a daily administration regimen is still considered effective. Additionally, the role of surgical treatment is evaluated for patients with localized disease, particularly those with upper lobe cavitary disease and patients with macrolide-resistant MAC pulmonary disease. It is suggested that patients receive intravenous amikacin three times per week for 6-8 weeks before surgery and four weeks after surgery.

The treatment duration is determined based on chest radiology and microbiologic test results during follow-up. Although it is usually recommended to continue treatment for a year after a negative microbiological test (86), there is limited treatment duration data from patients with extrapulmonary and disseminated disease. In cases with immunosuppression from anti-interferon gamma, it is suggested to continue treatment for 2–4 years, along with evidence of improved clinical symptoms, no detected lymphadenopathy, no new infiltration in chest radiology, and negative microbiological tests. To prevent re-infection in patients with HIV and disseminate MAC infection, continuous treatment is recommended. Treatment can be stopped once there is improvement, and the patient has completed at least one year of treatment. CD4 levels should also be monitored for at least six months after treatment, and they should be >100 cells/mm^3^ (126). Notably, when considering other medicines that increase regimen efficiency, it is crucial to account for the relevant treatment phase. Although *in vitro* AST is not always feasible, regular AST or MIC testing is recommended before treatment initiation. For tailored regimens, it is also essential to consider comorbidities, treatment duration, and long-term adverse effects (86). The summary of the studies related to antimicrobials used for treating MAC infection is shown in **Table 5**.

**Table 5:** Summary of the clinical studies in alternative agents for *Mycobacterium avium* complex (MAC) treatments.

Drugs	Regimen	Study design	Population	Primary endpoint	Outcomes	Adverse effects	Comments	Author, Year [References]
Aminoglycosides	Inhaled amikacin addition to the antimycobacterial regimen	Observational study, n = 6	Patients’ intolerant to parenteral aminoglycosides or as an adjuvant to oral therapy	Culture conversion and symptom improvement	All of the responders (n = 5, 83%) achieved symptomatic improvement and four were sputum culture negative after 6 months of therapy.	No adverse effects related to treatment were reported. One died from progressive respiratory failure.	Small case number	Davis et al., 2007 (127).
	Inhaled parenteral amikacin, addition to the antimycobacterial regimen	Observational study, n = 20	As of 20 participants, 25% were refractory pulmonary MAC and 75% were *M. abscessus* infection with the characteristics of bronchiectasis (100%), cystic fibrosis (10%), and primary ciliary dyskinesia (5%).	Culture conversion, symptoms, and computed tomography scan changes	18-25% of patients achieved sustained negative cultures along with symptom scores improved in 45%, were unchanged in 35%, and worsened in 20%, and computed tomography scans improved in 30%, were unchanged in 15%, and worsened in 55%.	Adverse effects were common, in 35% of patients stopping the drug because of side effects, including ototoxicity (10%), hemoptysis (10%), and nephrotoxicity (5%), persistent dysphonia (5%), and vertigo (5%).	Small case number	Oliver, et al., 2014 (128).
	Inhaled parenteral amikacin, addition to the antimycobacterial regimen	77 patients with refractory nontuberculous mycobacterium (NTM) pulmonary disease, including *Mycobacterium abscessus* complex (*n* = 48, 62%), MAC (*n* = 20, 26%) and mixed infections (*n* = 9, 12%).	Initiated inhaled parenteral amikacin as a primary treatment in participants with macrolide resistance (n = 63, 82%) and baseline amikacin resistance (n = 5, 6%).	Culture conversion, symptom improvement	At 12 months after initiation of treatment, 49% reported symptomatic improvement and 42% had radiological improvement	Adverse effects were common, with 38% of patients reporting an adverse event, with ototoxicity (n = 15) being the most common.	No monthly sputum follow-up made it was not possible to report the accurate time to sputum culture conversion and short follow-up duration	Jhun et al., 2018 (129).
	Amikacin liposomal inhalation suspension (ALIS)	Phase-2, double-blinded, randomized trial, n = 89 (ALIS = 44; placebo = 45)	ALIS 590 mg or placebo once daily added to the multidrug regimen for 84 days in treatment-refractory pulmonary nontuberculous mycobacterial (MAC or *Mycobacterium abscessus*) disease.	Change from baseline to Day 84 on a semiquantitative mycobacterial growth scale.	The primary endpoint was not achieved (*p* = 0.072); however, a greater proportion of the LAI group demonstrated at least one negative sputum culture (14 [32%] of 44 vs. 4 [9%] of 45; *p* = 0.006). Improved six-minute-walk test performance (+20.6 m vs. -25.0 m; *p* = 0.017) at Day 84 in the ALIS-treated patients	There was a greater percentage of respiratory adverse events in ALIS group (43% of participants developed dysphonia, 39% developed bronchiectasis exacerbation, 32% developed coughing, 21% developed oropharyngeal pain, and 11% chest discomfort, and 16% discontinued therapy due to adverse events).	A treatment effect was seen predominantly in patients without cystic fibrosis with MAC and was sustained 1 year after ALIS administration.	Oliver, et al., 2017 (130).
	Amikacin liposomal inhalation suspension (ALIS)	Phase-3, 2:1 open-label randomized trial (CONVERT), n = 336 (ALIS + GBT, *n* = 224; GBT-alone, *n* = 112)	ALIS 590 mg once daily was added to standard guideline-based therapy (GBT) in adults with amikacin-susceptible MAC lung disease and MAC-positive sputum cultures despite at least 6 months of stable GBT (224 vs. 112 participants). Notably, most had underlying bronchiectasis (62.5%), chronic obstructive pulmonary disease (14.3%), or both (11.9%).	Culture conversion (defined as three consecutive monthly MAC-negative sputum cultures by Month 6) Secondary endpoint: comparisons by treatment arm of change from baseline in the distance achieved in the 6-minute-walk test (6MWT), time to culture conversion, and change from baseline in St George’s Respiratory Questionnaire (SGRQ) scores.	Increased sputum conversion rates by the sixth month of treatment (29% in ALIS plus GBT) vs. 9% (GBT alone); odds ratio 4.22, [95% CI 2.08-8.57] and in 13.7% vs. 4.5% of participants with clarithromycin-resistant MAC isolates (MIC >32 mg/mL).	There was a higher rate of respiratory adverse events in ALIS group (46% of participants developed dysphonia, 37% developed coughing, 22% developed dyspnea, and 17% discontinued therapy due to adverse events).	Treatment outcomes were similar to those of an earlier phase 2 randomized trial in which the addition of ALIS to a standard multidrug regimen did not result in a greater reduction in mycobacterial growth on semi-quantitative sputum cultures compared with placebo but did not result in a higher proportion of at least one negative sputum culture (32 versus 9 percent). Regarding adverse events, the physician-guided measures (e.g., bronchodilator uses, oral rinses, and/or temporary dosing adjustments) may be useful resulted in symptomatic improvement.	Griffith et al., 2018 (131).
	Amikacin liposomal inhalation suspension (ALIS)	Retrospective cohort, n = 331	All-Payer Claims Database included MAC patients aged ≥ 18 years with ≥ 1 pharmacy claim for ALIS and ≥ 12 months of continuous health plan enrollment pre- and post-ALIS initiation.	Assessing changes in healthcare resource utilization (HCRU) among patients initiating ALIS, defined as respiratory disease-related (and all-cause) HCRU (hospitalization, length of stay, emergency department visits, and outpatient office visits) were compared 12 months pre- and post-ALIS initiation	Respiratory-related hospitalizations decreased to 19.3% (*p* < 0.01) and 15.4% (*p* < 0.0001) during 0–6 and 7–12 months post-ALIS initiation, respectively.		Significant reductions per patient/ 6-month period in all-cause and respiratory-related outpatient office visits were observed post-ALIS initiation (all *p* < 0.01).	Aksamit et al., 2022 (132).
Bedaquiline (BDQ)	BDQ 400 mg/day for 2 weeks then 200 mg ≥ 6 months PLUS ethambutol, rifampin (or rifabutin), and streptomycin or PLUS ethambutol, azithromycin (clarithromycin), amikacin, rifabutin, and streptomycin.	Case series, n = 6	Nodular bronchiectasis (n = 2), cavitary lung disease (n = 4) Macrolide susceptibility: resistance (n = 2), susceptible (n = 4)	Culture conversion and symptom improvement	Three had symptom improvement by six months and three had some improvement in semi-quantitative sputum culture.	Common side effects included nausea, arthralgias, anorexia and subjective fever.	This case series included patients with MAC lung disease who had been treated with multiple antibiotics over long time periods without success, which be supposed to be a group of notoriously difficult to treat effectively.	Philley et al., 2015 (122).
	BDQ 400 mg/day for 2 weeks then 200 mg ≥ 6 months PLUS ethambutol, rifampin (or rifabutin), and streptomycin or PLUS amikacin, ethambutol, and rifampin or PLUS ethambutol, azithromycin (clarithromycin), amikacin, rifabutin, and streptomycin.	Case series, n = 7	Fibrous nodular and cavitary (n = 3), nodular bronchiectasis and cavitary (n = 2), and nodular bronchiectasis (n = 2) Macrolide susceptibility: resistance (n = 3), susceptible (n = 4)	Culture conversion	After 6 months of treatment, 71% of participants (five of seven) had a microbiologic response, but all relapsed.		The emergence of *mmpT5* variants is associated with microbiological relapse following Mac treating with BDQ.	Alexander et al., 2017 (133).
	BDQ 400 mg/day for 2 weeks then 200 mg thrice weekly PLUS clofazimine 100 mg once daily + Clarithromycin 500 mg twice daily + ethambutol 1200 mg once daily	Case study, n = 1	Nodular bronchiectasis with heterozygous F508del *CFTR* gene mutation.	Outcomes (culture conversion, cure, symptom improvement) measured by 12 months	Culture positivity at Month 12 with persistent symptoms, BDQ was stopped and a bilobectomy was performed.		Drug-drug interaction and inadequate dose of BDQ may be a cause of treatment failure, particularly in treatment-refractory MAC pulmonary disease.	Zweijpfenning et al., 2019 (134).
	Azithromycin, rifabutin, and ciprofloxacin were initiated (ethambutol was excluded because of color blindness) PLUS tigecycline and linezolid, and, then, BDQ 18 months was initiated along with tedizolid	Case study, n = 1	Co-infection of MAC and HIV infection	Symptom improvement	Culture positivity at 12 months but symptom improvement at 18 months	No side-effect		Gil et al., 2021 (135).
Linezolid	Median linezolid therapy duration after the initial drug start was 21.4 weeks (range 1–201 weeks). 79% took 600 mg once daily, 12% took 300 mg once daily, and 5% took 600 mg twice daily.	Retrospective cohort study conducted at six NTM treatment centers in North America included 102 participants. Notably, 54% had pulmonary bronchiectasis, and 16% had COPD.	Common pathogens included *Mycobacterium abscessus* (44%), MAC (33%), and *Mycobacterium chelonae* (14%).	Tolerability of linezolid	Linezolid is well tolerated at a dosage of 600 mg/day.	40% of the patients had to stop taking linezolid. 46 patients (45%) experienced adverse events that their doctors attributed to linezolid after a median of 19.9 weeks (ranging from 0.1 to 107 weeks). Peripheral neuropathy developed in 24 patients (24%) within a median of 38 weeks (ranging from 2 to 233 weeks). Other adverse events attributed to linezolid included gastrointestinal intolerance (9%), anemia (8%), and thrombocytopenia (6%).	Has MIC_90_ values of <0.016 mcg/mL for MAC	Winthrop et al., 2015 (136).
Moxifloxacin	Macrolide-based three-drug regimen	41 patients with MAC refractory to a macrolide-based three drug regimen		Culture conversion	The overall treatment success rate was 29% (12/41), and the median time to sputum conversion was 91 days (IQR, 45 to 190 days).		Moxifloxacin has variable *in vitro* and *in vivo* activity against MAC Rifampin can reduce the serum concentration of moxifloxacin. However, combining quinolones with macrolides increases the risk of developing macrolide resistance.	Koh et al., 2013 (137).

ALIS, amikacin liposomal inhalation suspension; BDQ, bedaquiline; COPD, chronic pulmonary obstructive disease; GBT, guideline-based therapy; HIV, human immunodeficiency virus; HRCU, healthcare resource utilization; IQR, interquartile range; MIC, minimum inhibitory concentration; NTM, non-tuberculous mycobacterium; SGRQ, St. George’s Respiratory Questionnaire.

### 3.4 Monitoring drug–drug interactions

The use of medications for pre-existing conditions like diabetes mellitus, hypertension, hyperlipidemia, coronary artery disease, and hyperuricemia, is necessary during treatment for MAC infection. However, such medications can often interact with the antimycobacterial drugs used to treat NTM. These interactions can occur through various mechanisms, including competition for the cytochrome P450 (CYP) isoenzyme and P-glycoprotein, diminished absorption because of absorption competition with divalent ions, and the increased toxicity of combined medications. Notably, the extent of interaction depends on drug concentration, duration of administration, and the affinity of the drug and its metabolites. Critically, the prescription of CYP-based medications along with antimycobacterial drugs should be carefully considered and monitored. Generally, CYP inducers increase CYP substrate metabolism, thereby decreasing circulating substrate levels, while CYP inhibitors have the opposite effect.

Treatment for MAC infection can also lead to cardiovascular complications. Antimycobacterial drugs, including fluoroquinolones, macrolides, clofazimine, and bedaquiline are known to prolong the QTc interval and must be monitored closely to avoid arrhythmia-associated electrolyte and mineral imbalances, such as hypokalemia and hypomagnesemia (138), especially when used with loop diuretic or thiazides. Individuals who are co-administered trimethoprim/sulfamethoxazole should be made aware of its combination with potassium-sparing diuretics, such as spironolactone, angiotensin-converting enzyme inhibitors, angiotensin II-receptor blockers, and selective mineralocorticoid receptor antagonists (eplerenone and finerenone). Furthermore, if patients with diabetes mellitus take fluoroquinolone and oral hypoglycemic agents, they should be monitored for hypoglycemia because of fluoroquinolone’s ability to increase insulin release by blocking adenosine triphosphate-sensitive potassium channels in pancreatic β-cells (139). During treatment for MAC infection, several drug–drug interactions can cause severe organ injury. For example, the combined use of rifamycins, macrolides, and/or bedaquiline can cause hepatotoxicity (140), while combining trimethoprim/sulfamethoxazole with aminoglycosides can elevate the risk of acute kidney injury, as well as the risk of acute interstitial nephritis, which is caused by trimethoprim/sulfamethoxazole alone. In cases with liver and/or renal injury, antimycobacterial drug administration must be adjusted according to the daily dose.

Drug-drug interactions between medications used for MAC infection treatment and HIV infection treatment are a critical consideration due to the overlapping metabolic pathways and potential for adverse effects. For instance, rifabutin, a common MAC infection treatment, interacts with HIV protease inhibitors (PIs) like ritonavir, as ritonavir inhibits cytochrome P450 3A4 (CYP3A4), leading to increased rifabutin levels and a higher risk of toxicity (141). Similarly, clarithromycin, another MAC antibiotic, can increase concentrations of HIV non-nucleoside reverse transcriptase inhibitors (NNRTIs) like efavirenz due to CYP3A4 inhibition, potentially exacerbating CNS side effects (142). Conversely, azithromycin, often preferred over clarithromycin in HIV patients, has fewer CYP3A4 interactions but may still prolong the QT interval when combined with certain antiretrovirals like rilpivirine (126). Ethambutol, another MAC drug, generally has minimal interactions with antiretrovirals but may require dose adjustments when combined with nephrotoxic drugs like tenofovir disoproxil fumarate (TDF) due to additive renal toxicity (5). Lastly, fluconazole, sometimes used for MAC prophylaxis, can increase concentrations of HIV integrase strand transfer inhibitors (INSTIs) like dolutegravir by inhibiting CYP3A4 and UGT1A1, necessitating close monitoring (143). These examples underscore the importance of careful management and monitoring when co-administering MAC and HIV treatments to minimize adverse outcomes.

### 3.5 Multidisciplinary health personnel care team

The adoption of a multidisciplinary team approach is considered a critical factor for successful treatment for NTM. The team should be made up of several healthcare professionals, including pulmonologists, infectious disease physicians, intensivists, radiologists, microbiologists, respiratory therapy-specialized physical therapists, primary care physicians, and pathologists (144). Social workers, nutritionists, clinical pharmacists, and family support are also essential members of the team. Social workers play vital roles in assisting patients to cope with psychosocial and financial concerns (128), while clinical pharmacists provide thorough information on antimycobacterial dosing, drug interactions, and adverse events. For long-term treatments, therapeutic drug monitoring is becoming increasingly necessary to ensure optimal therapeutic levels and prevent unexpected adverse drug reactions. A multidisciplinary approach has numerous benefits, including excellent clinical outcomes and the prospect of future research for disease control and efficient treatment strategies. It is also worth noting that collaborative approaches ensure patient compliance and improve the provision of quality care services. Therefore, for successful NTM infection management, healthcare providers should embrace multidisciplinary team approaches as the standard of care.

### 3.6 Perspectives on non-tuberculous mycobacteria infection

Although NTM virulence is relatively less than that of *M. tuberculosis* infection, as indicated by typical self-limiting disease, anti-NTB host immune responses induce lung injury, which might advance the disease or cause its insidious progression. NTM can normally be found in the airways of <1% of healthy hosts. However, NTM might interact with other bacteria in the host’s microenvironment, and changes in NTB numbers might affect other bacteria and *vice versa*. Interestingly, non-NTM pulmonary microbiota populations might affect treatment outcomes. Moreover, the intestines, which have high levels of microorganisms, have several mechanisms of communication with other organs, including the lungs (referred to as the “gut–lung axis”), which might affect lung treatment outcomes, and the intestines have an easier sample collection procedure (via feces) when compared with lungs (bronchoalveolar lavage). Based on the microbial signature of the lungs and gut, interventions that alter both microbiota might be a future treatment for NTM strategy as part of personalized or designer medicine.

## 4 Conclusions

NTM infection diagnosis and treatment require a combination of scientific and artistic approaches. NTM’s insidious onset and predominantly asymptomatic features in the early disease stages can pose challenges to timely detection and diagnosis. Thus, individuals with preexisting risk factors, including older individuals, those with bronchiectasis, and those with immunosuppression warrant greater attention. Moreover, a comprehensive understanding of the underlying pathogenesis and potential complications can prevent unfavorable outcomes and improve prognosis. Recent evidence has linked gut pathogen homeostasis imbalance to disease progression. Effective NTM management is often based on the interpretation of susceptibility breakpoints and a multidisciplinary approach, which is imperative for ensuring holistic, patient-centric care that fosters good compliance with long-term treatment.
